# Optimized electroacupuncture treatment for female stress urinary incontinence: study protocol for a multi-center randomized controlled trial

**DOI:** 10.3389/fpsyt.2023.1228131

**Published:** 2023-08-17

**Authors:** Lumin Liu, Bingli Chen, Xiaohui Si, Wenguang Hou, Qian Fan, Xu Li, Juanjuan Li, Shuren Ming, Ping Yin, Yuelai Chen

**Affiliations:** ^1^LongHua Hospital Shanghai University of Traditional Chinese Medicine, Shanghai, China; ^2^Yueyang Hospital of Integrated Traditional Chinese and Western Medicine, Shanghai University of Traditional Chinese Medicine, Shanghai, China

**Keywords:** electroacupuncture, stress urinary incontinence, randomized clinical trial, protocol, female

## Abstract

**Background:**

Stress urinary incontinence (SUI) is a common condition that can severely affect women’s life quality. Electroacupuncture (EA) has been proved to be an optional treatment for SUI, but the tolerance of EA becomes a factor affecting efficiency, which should not be ignored and needs to be solved urgently. The purpose of this study is to find out whether the use of alternating acupoints combination can solve this problem or not and provide an optimization of EA treatment for female SUI.

**Methods:**

This multi-center randomized controlled trial will enroll 360 patients with SUI. They will be randomly assigned to one of the three groups—sacral acupoints group (sacral group), abdominal acupoints group (abdominal group), or alternating acupoints group (alternating group)—at a 1:1:1 ratio. The patients will receive 18 sessions of EA treatment and will be followed up for 48 weeks after the treatment. The primary outcome measure of the study is the change of urine leakage at week 6. The secondary outcomes include the incontinence episode frequency (IEF), International Consultation on Incontinence Questionnaire-Short Form (ICIQ-SF), severity of SUI, patient self-evaluation of therapeutic effects, weekly usage of urine pads, ultrasonography of pelvic floor, specialty therapies for SUI, evaluation of discomfort during EA treatment, patient acceptability evaluation and adverse events related to intervention.

**Discussion:**

This trial is specifically designed to offer an optimized EA treatment for female SUI, aiming to enhance their quality of life.

**Clinical trial registration**: ClinicalTrials.gov, identifier ID:NCT05635669.

## Background and objective

Stress urinary incontinence (SUI) mainly refers to the involuntary leakage of urine when abdominal pressure increases, such as sneezing, coughing, laughing or exercise (1). According to epidemiological studies, the global prevalence of female SUI is 20%–45% (2, 3). A longitudinal study among adult women in China has found that the incidence of female SUI is 13.1 per 1,000 person-years. As the prevalence of SUI increases with age, it will still be on the rise with the aging process of China’s population (4). The clinical symptoms of SUI not only affect patients’ mental health and life quality (5), but also cause an economic burden to society (6). Thus, more attention should be paid to this public health concern.

At present, the treatment of SUI includes behavioral therapy, physiotherapy, medications, devices and surgery, among which pelvic floor muscle training (PFMT) is the most preferred treatment for patients with mild to moderate SUI (7). However, many studies have found that PFMT requires long-term adherence for at least 3 months, which is a big challenge for patients’ compliance and time (8). Therefore, the efficacy of PFMT in the treatment of SUI has not met expectations.

To solve the unmet clinical need for SUI treatment, acupuncture is gradually widely used. A previous study has demonstrated that electroacupuncture (EA) intervention can reduce the urine leakage in pad test and decrease the International Consultation on Incontinence Questionnaire-Short Form (ICIQ-SF) score, which can help patients alleviate symptoms and improve their life quality (9). However, we have found that EA tolerance may has an impact on the efficacy in SUI treatment. The curative effect in the early stage may be more obvious than that in the later due to the repeated stimulation of a certain acupoint or acupoints combination. Based on our clinical experience, the alternate use of acupoint or acupoints combination can solve this problem. Hence, this trial is designed to explore a relatively suitable application of acupoints combination and provide certain clinical evidence for the optimization of EA in treating female SUI.

## Methods

### Study design

**Figure 1:**
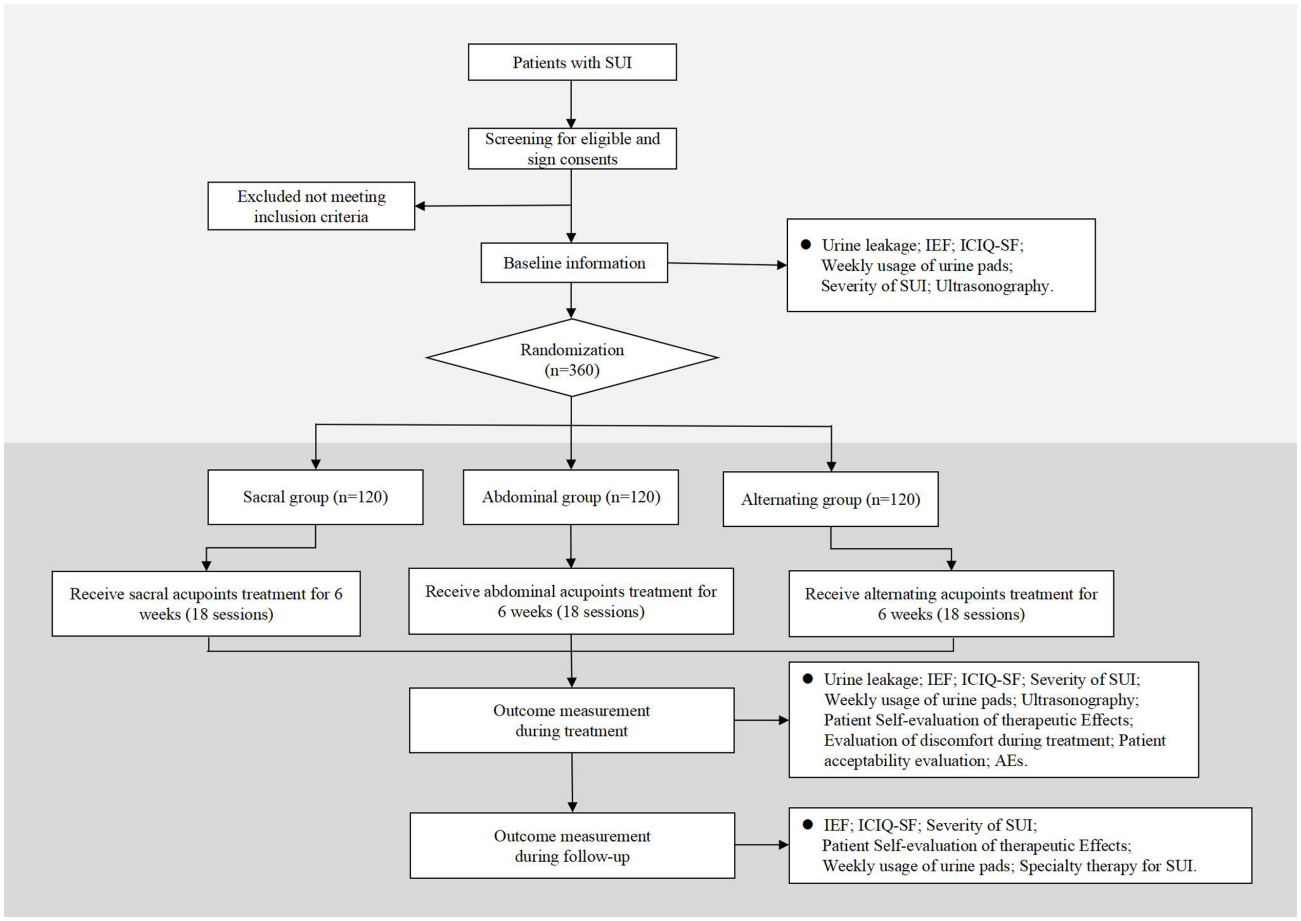
Flowchart. SUI, stress urinary incontinence; IEF, incontinence episode frequency; ICIQ-SF, International Consultation on Incontinence Questionnaire-Short Form; AEs, adverse events.

This multi-center, randomized, controlled trial will be conducted at LongHua Hospital Shanghai University of Traditional Chinese Medicine, Obstetrics and Gynecology Hospital of Fudan University, Yueyang Hospital of Integrated Traditional Chinese and Western Medicine of Shanghai University of Traditional Chinese Medicine, and Seventh People’s Hospital. It has been approved by the Medical Ethics Committee of LongHua Hospital Shanghai University of Traditional Chinese Medicine (No. 2022LCSY084) and registered at ClinicalTrials Registry (NCT05635669). This protocol is reported based on Recommendations for Interventional Trials (SPIRIT) 2013 Checklist. Any amendment of the protocol will be recorded and reported. Women with SUI will be randomized into sacral group, abdominal group or alternating group at a 1:1:1 ratio. The flowchart of the trail is shown in Figure 1 while the schedule of enrollment, interventions, and assessment is presented in Table 1.

**Table 1:** Schedule of recruitment, interventions and assessments.

Study period	Enrolment		Intervention						Follow-up			
Time point	Week −1	Week 0	Week 1	Week 2	Week 3	Week 4	Week 5	Week 6	Week 18	Week 30	Week 42	Week 54
Inclusion/exclusion Criteria	**X**											
Sign informed consent	**X**											
Randomization	**X**											
*Intervention*												
Sacral acupoints treatment			**X**	**X**	**X**	**X**	**X**	**X**				
Abdominal acupoints treatment			**X**	**X**	**X**	**X**	**X**	**X**				
Alternating acupoints treatment			**X**	**X**	**X**	**X**	**X**	**X**				
*Primary outcome*												
Urine leakage		**X**						**X**				
*Secondary outcomes*												
Incontinence episode frequency		**X**				**X**		**X**	**X**	**X**	**X**	**X**
ICIQ-SF		**X**				**X**		**X**	**X**	**X**	**X**	**X**
Severity of SUI		**X**				**X**		**X**	**X**	**X**	**X**	**X**
Patient self-evaluation of therapeutic effects						**X**		**X**	**X**	**X**	**X**	**X**
Weekly usage of urine pads		**X**				**X**		**X**	**X**	**X**	**X**	**X**
Ultrasonography		**X**						**X**				
Specialty therapy for SUI									**X**	**X**	**X**	**X**
Evaluation of discomfort during treatment			**X**		**X**							
Patient acceptability evaluation			**X**		**X**							
Safety evaluation			**X**	**X**	**X**	**X**	**X**	**X**				

SUI, stress urinary incontinence; ICIQ-SF, International Consultation on Incontinence Questionnaire-Short Form.

### Patient recruitment

Eligible patients with SUI will be recruited in outpatient clinics from the four participating hospitals. And those patients will be firstly assessed for eligibility by trained personnel face-to-face following the inclusion and exclusion criteria mentioned below. Before randomization, written informed consent will be obtained from all patients. They have the right to withdraw at any time and their personal data will only be used for this study.

### Criteria for diagnosis

Patients must meet the following criteria of SUI (10):

1. Involuntary loss of urine with physical exertion or effort, coughing or sneezing;
2. 1 h urine pad test indicating weight gain >1 g;
3. Absence of other symptoms of urinary frequency or urgency.

The severity of SUI is distinguished according to the 1 h urine pad test (11): normal: ≤ 1 g; mild: 1.1 g–9.9 g; moderate: 10 g–49.9 g; severe ≥50 g.

### Criteria for inclusion

1. Patients with mild to moderate SUI mentioned in the diagnostic criteria above;
2. Female, aged 40–75 years;
3. Sign the informed consent.

### Criteria for exclusion

1. Other types of urinary incontinence (urgent, overflow or mixed);
2. History of urinary incontinence surgery or pelvic floor surgery;
3. Pelvic organ prolapse ≥stage II;
4. Symptomatic urinary tract infection;
5. Residual urine volume >30 mL;
6. Maximum urinary flow rate <20 mL/s.
7. Limitation of movement (walk and/or run and/or climb stairs);
8. Patients who have been using drugs that may affect bladder function or have been receiving SUI specialized treatment;
9. Severe cardiovascular, cerebral, liver, kidney and hematopoietic system disease, mental disorders, diabetes, multiple system atrophy, cauda equina neuropathy and spinal cord disease;
10. Pregnancy or lactation period;
11. With cardiac pacemaker, EA phobia or metal allergies.

### Criteria for removal

1. Patients who did not complete the full course of 18 sessions over 6 weeks and did not provide a primary outcome indicator after treatment.
2. Patients with the above exclusion criteria occurring during treatment;
3. Voluntary patient withdrawal from study treatment;
4. Occurrence of severe adverse events (AEs) or complications that result in stopping the trial.

### Randomization, allocation concealment and blinding

The random assignment scheme for this study was generated by the Clinical Research Center of LongHua Hospital Shanghai University of Traditional Chinese Medicine with “Proc plan” program of SAS 9.4 (SAS Institute, Cary, NC, United States). In this study, stratified randomization was adopted, with the research centers as the stratification factor. All the 4 participating hospitals will compete for enrollment. The random distribution cards sealed in opaque envelopes will be opened by the personnel for grouping according to the sequence of hospital visits so that patients can be assigned to sacral group, abdominal group or alternating group at 1:1:1 ratio. Based on the characteristics of this study, assessors for information and statisticians are blinded. Moreover, all researchers who have received training on the implementation of this research will strictly abide by the principle of departments separation.

### Interventions

All treatments will be performed after skin disinfection. As acupuncture needles are inserted, all needles will be lifted, twisted, and stabbed to activate the sensation of “de qi.” The EA stimulation will last for 30 min with a continuous wave of 50 Hz and a current intensity of 1 to 5 mA (12). In the study, sterile acupuncture needles will be used, which are from Suzhou Medical Supplies Factory Co.’s Huatuo (size 0.30 × 75 mm). Patients will receive 3 treatments per week (every other day), for a total of 18 sessions over the course of 6 weeks. The follow-up sessions are in weeks 18, 30, 42 and 54. Besides, patients are told that they cannot use specialty therapy for SUI, such as pelvic floor muscle training, electrical stimulation, biofeedback, vaginal cone and medication during the 6 weeks treatment period.

### Sacral acupoints group (sacral group)

**Figure 2:**
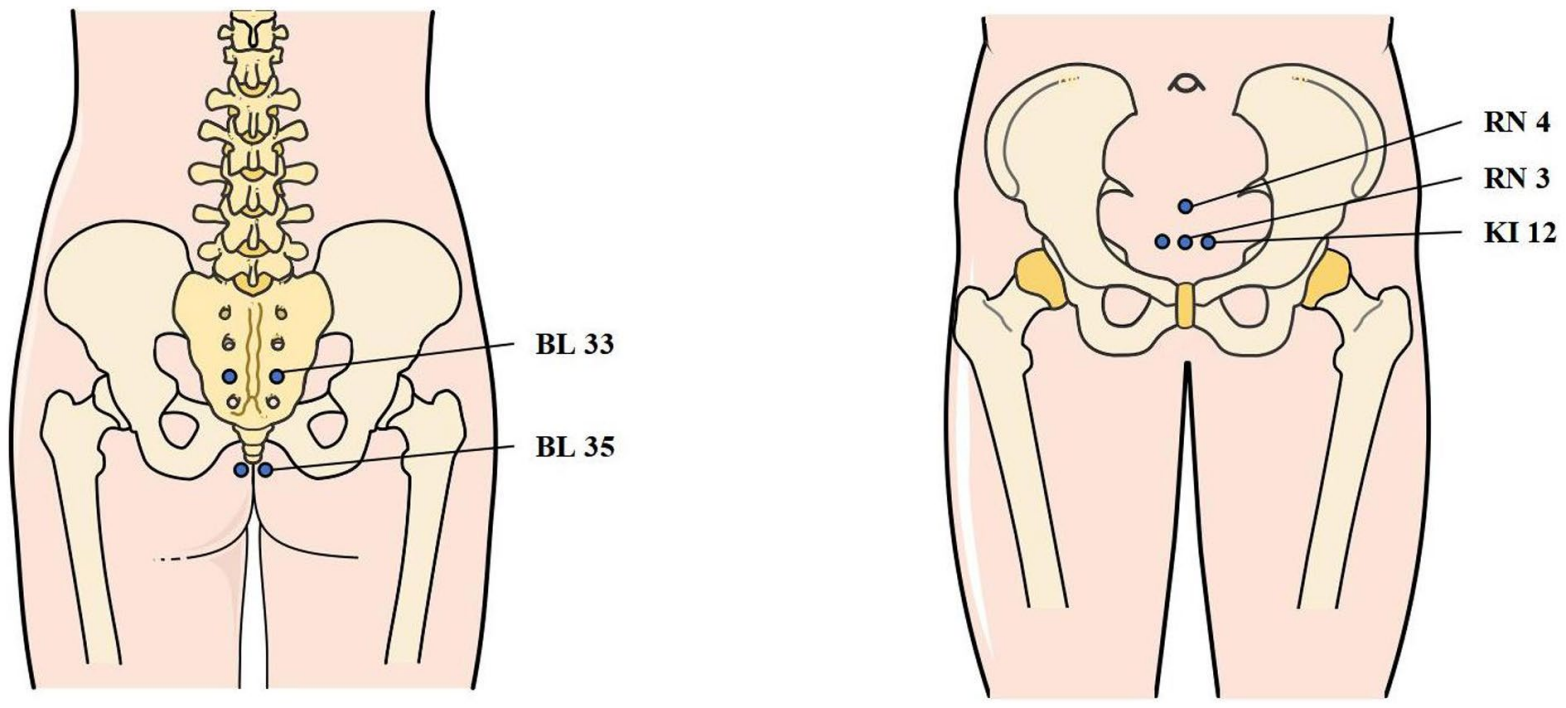
Locations of acupoints. BL33: Zhong Liao; BL35: Hui Yang; RN3: Zhong Ji; RN4: Guan Yuan; KI12: Da He.

Patients will receive EA treatment of sacral acupoints combination treatment at bilaterally Huiyang (BL35) and Zhongliao (BL33) in the prone position during the whole treatment course. In this group, paired electrodes from the EA apparatus will be attached transversely to the needle handles at bilateral BL35 and BL33. The acupoints’ locations and manipulating techniques are described in Figure 2 and Table 2.

**Table 2:** Locations and manipulations of acupoints.

Acupoint	Location	Manipulation
Zhong Ji (RN3)	4 cun below the navel, on the anterior midline	Needle insertion followed an angle of 30°to 45° towards perineum
Guan Yuan (RN4)	3 cun below the navel, on the anterior midline	Needle insertion followed an angle of 30°to 45° towards perineum
Da He (KI12)	4 cun below the navel, 0.5 cun lateral to the anterior midline	Needle insertion followed an angle of 30°to 45° towards perineum
Zhong Liao (BL33)	located in the third sacral foramen	Needle insertion followed an angle of 30° to 45° in an inferomedial direction
Hui Yang (BL35)	located 0.5 cun lateral to the extremity of the coccyx	Needle insertion followed an angle of 30° to 45° in a slightly superolateral direction

### Abdominal acupoints group (abdominal group)

Patients will receive EA treatment of abdominal acupoints combination at Zhongji (RN3), Guanyuan (RN4) and bilaterally Dahe (KI12) in the dorsal position during the whole treatment course. In this group, paired electrodes from the EA apparatus will be attached to the needle handles, respectively, at RN3 and left side of KI12, as well as RN4 and right side of KI12. The acupoints’ locations and manipulating techniques are described in Figure 2 and Table 2.

### Alternating acupoints group (alternating group)

Patients will receive EA treatment of sacral acupoints combination and abdominal acupoints combination alternately. (For example, A for the first time, B for the second time, A for the third time, and so on). The application of EA is the same as that of sacral group and abdominal group.

## Outcomes

The primary outcome of the study will be the change of urine leakage between baseline and week 6 measured by the 1 h pad test. The standardized procedure of the 1 h pad test requires the patients to wear a pre-weighed pad, drink 500 mL of water within 15 min, and then complete various activities including walking, going up and down stairs, standing and sitting, picking up a coin from the floor, coughing, running, and putting their hands under running water. Afterwards, the pad will be reweighed to measure the amount of urinary leakage (13). The 1 h pad test has a high adherence rate and a specificity of 65% to 89% (14). When patients have periods or a severe cough, outcome evaluations will be postponed and rescheduled until the end of the period or the remission of the cough.

The secondary outcomes include the following ten items:

1. The change of mean incontinence episode frequency (IEF) in 72 h.
2. The change of International Consultation on Incontinence Questionnaire-Short Form (ICIQ-SF). It is a brief instrument used to assess the IEF or the volume of incontinence, as well as the impact of life quality during the previous four weeks. We will utilize the Chinese version due to its characteristics of a patient-centered life quality assessment tool. This version is easy to understand and has good internal consistency and reliability (15). Scores range from 0 to 21, with higher numbers denoting more severity (16).
3. Severity of SUI. It is recorded by patients in 72 h bladder diaries. The following will be the levels of leakage: mild (a few drops), moderate (leak that soaked through underpants), and severe (leak that soaked through outerwear). If patients wear urinal pads, the severity of SUI will be assessed accordingly. Mild (few drops of leakage), moderate (soaked urine pads in patches by several leakages), and severe (soaked urine pads in patches by one leakage). The most extreme level of urine leakage will be used as the severity of SUI for analysis (12).
4. Patient self-evaluation of therapeutic effects. It will be observed with 0 to 3 indicating “no help,” “small help,” “medium help” and “great help” respectively (12).
5. Weekly usage of urine pads. Calculation methods at different time points will be as follows: weekly consumption of urine pads during weeks 1–6 equals the total of urine pads consumed during weeks 1–6 divided by 6; while the weekly consumption of urine pads during weeks 7–18 equals the total of urine pads consumed during weeks 7–18 divided by 12 (12).
6. The change of ultrasonography of pelvic floor from baseline to week 6. The indicators include bladder neck mobility, urethral rotation angle, rectovesical angle, lowest point of bladder, urethral funnel formation, the type of bladder bulging both at rest and during a Valsalva maneuver by ultrasonography (17). Thirty patients in each group will be randomly selected for this examination.
7. Specialty therapy for SUI. Application of specialty therapy for SUI will be observed at follow-up (weeks 18, 30, 42, 54). The number of patients using the specialty therapy and the frequency of treatment will be recorded.
8. Correlation analysis. The correlation between 1 h pad test and severity of SUI in week 6. The correlation between IEF in 72 h and severity of SUI in weeks 18, 30, 42, and 54.
9. Evaluation of discomfort during EA treatment: Visual analog scale (VAS) will be used to evaluate the degrees of discomfort during treatment. The scale ranges from 0 to 10 cm, with 0 cm indicating no discomfort and 10 cm indicating severe discomfort. The average value of the first and ninth acupuncture treatments will be taken within 5 min after the end of the treatments. If the VAS value is missing in one of the cases, another will be taken as the result.
10. Patient acceptability evaluation: 0 = very difficult to accept, 1 = slightly difficult to accept, 2 = acceptable, 3 = easy to accept, 4 = very easy to accept. The average value of the first and ninth acupuncture treatments will be taken within 5 min after the end of the two treatments. If the VAS value is missing in one of the cases, another will be taken as the result.

The time point of measures 1.2.3.4.5 will be in weeks 4, 6, 18, 30, 42, and 54.

### Safety evaluation

AEs will be documented by researchers in case report form (CRF), including the date of onset and recovery, severity, relevance to the treatment, and how the AEs are resolved (or not). Treatment related AEs include dizziness, abscess, subcutaneous hematoma, local infection and other discomforts (i.e., fatigue, dizziness, or palpitations). If AEs do occur, the researchers will treat them appropriately, and notify the board who oversee data and safety of any severe ones. Then the board will assess whether the AEs are connected to the treatment and decide whether the study should be terminated or not. Frequency and percentage of patients suffering from AEs in three groups will be calculated.

### Sample size calculation

The PASS software (version 15.0.5, NCSS, LLC) was used to calculate sample size with *α* of 0.05 (two-tailed), power of 80%, and ratio of 1:1:1. Based on our previous research, the change of urine leakage between baseline and week 6 was calculated. For the sacral, abdominal, and alternating groups, the mean ± standard deviation (SD) was 9.90 ± 2.61, 10.30 ± 3.60 and 11.60 ± 3.93, respectively (12). Assuming a 20% dropout rate, the sample size is 120 patients for each group, and 360 patients in total.

### Statistical methods

Statistical analysis, including a full analysis set based on intent-to-treat principle and a per-protocol set, will be conducted with SAS 9.4. Continuous variables described as mean ± SD or median (interquartile range) will be analyzed by *t*-test or rank-sum test while categorical variables represented by frequency (composition ratio) will be analyzed by chi-square or Fisher’s exact tests. Generalized estimating equation or mixed effect model is suitable for trends over time and treatment-time interactions. The analysis of correlation will be conducted with spearman correlation analysis. Exploratory subgroup analysis will be conducted to explore possible sources (i.e., parity status, the duration after the last childbirth, etc.) of heterogeneity and inconsistency. The confidence interval will be set at 95%, with a significance level of 5% (*p* < 0.05).

### Quality control, data management and monitoring

Prior to the study, researchers will receive training on the protocol, the standard operating procedures, and the scale evaluation in order to increase the validity of clinical research. The acupuncture therapist for this study must be a licensed acupuncturist with at least 5 years’ clinical experience. Prior to the trial, the acupuncturist will receive rigorous standardized training on acupuncture point positioning, acupuncture technique specifications, and how to use the EA equipment. Moreover, a detailed data management plan has been set, including the collection, entry, and management of data. Data collected by CRF will be entered and confirmed independently by two researchers in order to guarantee the consistency. In addition, study progress and data quality checks will be made by research assistants and monitors.

## Discussion

SUI seriously affects patients’ daily activities, sleep quality and social interactions, decreasing their life quality and causing negative emotions such as anxiety and depression due to the worry caused by leakage and the use of urine pads (18). The efficacy of EA for SUI has been widely recognized (9). In previous studies of multi-center, large-sample, randomized controlled trials, it has been demonstrated that SUI can be relieved or cured by EA treatment of sacral acupoints (BL33 and BL35) and abdominal ones (RN3, RN4 and KI12) (12, 19, 20).

However, we have found a clinical phenomenon that some patients had better curative effect in the early stage of the treatment than that in the later one. It might be because the body changed from sensitivity to adaptation and thus EA tolerance occurred after the same acupoint was repeatedly stimulated. Studies have shown that rats’ alteration of tail flick latency in EA treatment was higher from day 1 to day 5, but did not differ from the sham treatment from day 6 to day 8 (21); in addition, the percentage change in tail flick latency reached 61.4 ± 5.5% at day 1 but decreased to 31.3 ± 7.9% at day 3 and to 2.1 ± 4.1% at day 8 (22). The researches mentioned above have provided evidence that the phenomenon of EA tolerance is objective and related to EA efficiency.

The mechanisms of EA tolerance are mainly related to peripheral and central parts. For the peripheral one, receptors at the acupoint can receive stimulation, but repeated stimulation makes the receptors less sensitive and more tolerable (23). In the central nervous system, EA has been demonstrated to be able to motivate the release of opioids, and there are parallels between the mechanisms of EA tolerance and opioid tolerance (24). Repeated EA stimulation results in an excessive release of anti-opioid substances (e.g., cholecystokinin octapeptide, orphanin FQ, etc.) or a decreased number of opioid receptors, thereby reducing the original effect and contributing to EA tolerance (25–27).

It can be seen that the EA tolerance might be a key factor affecting the efficacy in EA treatment, but it has been rarely reported. Given that repeated stimulation of the same acupoints may lead to EA tolerance, we envisioned to provide treatments with alternating use of sacral and abdominal acupoints, which avoid the need to use the same set of acupoints every time. By adopting this approach, we aim to prevent EA tolerance caused by repeated stimulation, thus improving the clinical efficacy of the treatment.

In order to confirm the advantages of the treatment with alternating acupoints combination, a multi-center, stratified randomized study and a 48 weeks long-term follow-up with rigorous methodological design will be used to make the results more objective. In this study, the three groups will all receive acupuncture treatments (abdominal acupoints EA treatment, sacral acupoints EA treatment or alternating acupoints EA treatment), which encourage a better compliance of patients. On the other hand, we select BL33 and BL35 for the sacral group and RN3, RN4 and KI12 for the abdominal group. This is because our team’s previous investigations have demonstrated the effectiveness of EA using these acupoints in treating SUI (12, 19, 20). The positive outcomes may be attributed to EA’s capacity to address bladder neck and urethral support defects, modulate urinary bladder pressure, enhance urethral sphincter muscle tone, and reduce muscle fatigue (28–30). What’s more, the recovery of pelvic floor function is helpful for the treatment of SUI. Therefore, in addition to the use of the 1 h urinary pad test with standardized procedures, this study also used pelvic floor ultrasound for objective assessment of efficacy. At last, for medical ethical considerations, patients are allowed to receive specialty therapy for SUI during the follow-up period. The data will be collected as part of the secondary outcome, allowing us to compare the number of patients and frequency of use between the two groups. This analysis will not only offer insights into treatment effectiveness but also assess any potential sustained effects.

However, this study also has some limitations. Firstly, all the 4 medical centers participating in this study are all located in the same city, which may lead to the lack of diverse regions subjects. Diverse regions such as urban and rural areas may present varying prevalence rates, causes, lifestyles, and treatment responses related to SUI. To address this concern, we will recruit participants from four large, comprehensive tertiary hospitals, with block randomization and competing enrollment, aiming to enhance the sample’s diversity. Secondly, due to all three groups being treatment groups, the acupuncturists need to perform specific maneuvers to elicit the “de qi” sensation of “swelling, soreness, numbness, and heaviness” in patients. Therefore, the study is not double-blinded, potentially hindering the exclusion of the placebo effect of acupuncture. However, it is important to note that previous studies have compared EA with placebo EA, confirming that its efficacy in addressing SUI is not attributable to a placebo effect (12). Thus, this particular concern has been mitigated. In conclusion, we hope that the results of this study will provide an optimized EA treatment for female SUI, and ultimately aiming to enhance their quality of life.

## Trial status

The date of the intended trial period is from December 2022 to March 2025. Patients’ recruitment started in January 2023.

## Ethics statement

The studies involving humans were approved by Medical Ethics Committee of LongHua Hospital Shanghai University of Traditional Chinese Medicine. The studies were conducted in accordance with the local legislation and institutional requirements. The participants provided their written informed consent to participate in this study.

## Author contributions

YC and PY conceived the study and designed the study protocol. BC and XS made the first draft of the manuscript. LL and QF were responsible for the final manuscript. XL and JL participated in creating the statistical analysis plan. WH and SM contributed to the study design and made critical revisions. All authors read and approved the final manuscript.

## Funding

This work was supported by the Shanghai 2022 “Science and Technology Innovation Action Plan” Medical Innovation Research Special Project, with grant number (22Y21920100), the Shanghai Three-year Action Plan of Accelerating The Inheritance and Development of TCM—The Construction of TCM Specialty Alliance in East China Region and at The Municipal Level—The TCM Pelvic Floor Disease Rehabilitation Specialty Alliance Project (the grant number: ZY (2021-2023)-0302), the Shanghai Shenkang Hospital Development Center Medical Enterprise Integration Innovation Synergy Special Project (the grant number: SHDC2022CRT003), and Shanghai Famous Old Chinese Medicine Experts Academic Experience Research Studio Construction Project (SHGZS-202232), and Shanghai Shenkang Center, Construction of Demonstrative and Research-oriented Ward (SHDC2022CRW006). These funding sources played no part in study design and will not be involved in data execution, analyses, or interpretation.

## Conflict of interest

The authors declare that the research was conducted in the absence of any commercial or financial relationships that could be construed as a potential conflict of interest.

## Publisher’s note

All claims expressed in this article are solely those of the authors and do not necessarily represent those of their affiliated organizations, or those of the publisher, the editors and the reviewers. Any product that may be evaluated in this article, or claim that may be made by its manufacturer, is not guaranteed or endorsed by the publisher.

## Abbreviations

SUI: Stress urinary incontinence
EA: Electroacupuncture
IEF: Incontinence episode frequency
ICIQ-SF: International Consultation on Incontinence Questionnaire-Short Form
PFMT: Pelvic floor muscle training
AEs: Adverse events
SD: Standard deviation
CI: Confidence interval
